# Early-life gut microbial colonization shapes Th1/Th2 balance in asthma model in BALB/c mice

**DOI:** 10.1186/s12866-017-1044-0

**Published:** 2017-06-17

**Authors:** Li-Juan Qian, Shu-Min Kang, Jia-Li Xie, Li Huang, Quan Wen, Yuan-Yuan Fan, Li-Jun Lu, Li Jiang

**Affiliations:** 1 Department of Pediatrics, Zhongda Hospital, Southeast University, 87 Dingjiaqiao, Gulou District, Nanjing, Jiangsu 210009 China; 2 Department of Pediatrics, Zhangjiagang First Peoples Hospital, Suzhou, Jiangsu 100142 China

**Keywords:** Asthma, Early life, Diverse microbial environments, Gut microbial colonization, Th1/Th2 balance

## Abstract

**Background:**

We aimed to investigate the effect of early-life diverse microbial exposures on gut microbial colonization in an OVA-induced asthma model in BALB/c mice.

**Methods:**

BALB/c mice were divided into 4 groups: A, offsprings were kept in a SPF environment during fetal, lactation, and childhood periods; B, offsprings were kept in the SPF environment during fetal and lactation periods, and kept in the general environment during childhood; C, offsprings were kept in the SPF environment only during fetal period, and then kept in the general environment; and D, offsprings were kept in the general environment during whole periods. The diversity of intestinal flora was analyzed using denaturing gradient gel electrophoresis. Mice were sensitized with OVA to establish an animal model of asthma. Then asthma-related inflammatory cytokines and histological analysis were performed.

**Results:**

The diversity of intestinal microflora in group D was significantly higher than groups A, B and C at three days and three weeks after birth, and the diversity of intestinal microflora in groups C and D were significantly higher than groups A and B at five weeks after birth. The pathologic scores of OVA-induced asthmatic mice in group D were significantly lower than group A, and serum IFN-γ levels and the IFN-γ/IL-4 ratio in group D were significantly higher than group A.

**Conclusions:**

Exposure to diverse microbial environments in early life affects gut microbial colonization in BALB/c mice. The diversity of the intestinal flora in early life may prevent airway inflammation in asthma via regulating the Th1/Th2 balance.

## Highlights

1. Early-life diverse microbial exposures may affect gut microbial colonization.
2. Early-life diverse microbial exposures may affect Th1/Th2 balance.
3. Early-life diverse microbial exposures may prevent airway inflammation in asthma.

## Background

Asthma is a chronic airway disorder that is characterized by increased airway responsiveness and inflammation [1]. It is estimated that asthma affects approximately 300 million people worldwide [2], and its prevalence is steadily increasing [3]. Asthma is caused by complicated risk factors, including genetic variations and environmental factors [4].

Recently, multiple epidemiological studies have suggested that a more “westernized” life style, which is defined as a cleaner environment with reduced bacterial exposure, increases the prevalence of asthma [5]. Exposure to diverse microbes can influence airway microbial colonization or the composition of the gut microbiome, which can affect asthma development [6]. A previous study has demonstrated that the prevalence of asthma is lower in children living on farms as a result of diverse microbial exposure [7]. In addition, lower intestinal microbial diversity at one month of age is shown to be a potential predictor of atopic eczema at two years of age [8]. Reduced intestinal microbial diversity during infancy is likely to contribute to an increased risk of lifestyle-related disorders, such as allergic disease [9]. A meta-analysis of the association between childhood asthma and caesarean section also shows that the risk of childhood asthma may increase after cesarean section because this type of delivery protects an infant against early exposure to maternal gut and vaginal flora [10]. However, whether childhood asthma can be prevented via early exposure to diverse microbial environment to regulate the gut microbiome is unclear and merits further investigation.

Therefore, in the present study, we aimed to modulate early-life exposure to microbial environment to alter the gut microbiome, and explore the key mechanism involved in early-life gut microbial colonization in BALB/c mice. Our findings will help to devise approaches to prevent asthma.

## Methods

### Animals and grouping

Twenty-four adult specific pathogen-free (SPF) BALB/c mice (8 male and 16 female) were used in this study. Male and female mice were caged 2:1 for conception. Then, parents and their offsprings that reared in either a SPF environment or a general (microbially diverse) environment were divided into 4 groups according to different time periods, including the fetal period, lactation and childhood, as follows: Group A, offsprings were kept in a SPF environment during the fetal, lactation, and childhood time periods; Group B, offsprings were kept in the SPF environment during the fetal and lactation time periods, and then were kept in the general environment during childhood; Group C, offsprings were kept in the SPF environment during the fetal period, and then kept in the general environment during the lactation and childhood time periods; and Group D, offsprings were kept in the general environment during the fetal, lactation, and childhood time periods. For the SPF environment, air was highly filtered, food was irradiated (5 M Rad Co-60 for 20 h), and water and litter were autoclaved for obtaining an antigen-free environment. For the general environment, air was not highly filtered and the litter was not autoclaved. All mice were maintained in an incubator with controlled temperature (23 ± 1 °C) and humidity (60 ± 5%) under a 12-h light/dark cycle. Fecal samples were collected from these mice at three days, three weeks and five weeks after birth for subsequent analysis. All animal protocols were approved by the local Animal Care and Use Committee.

### Extraction of bacterial DNA from feces

Genomic DNA was extracted from feces as previous described [11] with some modifications. Fecal samples were homogenized in extraction buffer [50 mM Tris (pH 7.4), 100 mM EDTA (pH 8.0), 400 mM NaCl, and 0.5% SDS] supplemented with 20 μL of proteinase K (20 mg/mL), then mixed with zirconia/silica beads (500 μL, 0.1-mm diameter; BioSpec Products, Bartlesville, OK, USA) and placed in a MiniBeadbeater-8 cell disrupter (BioSpec Products) for 5 min. After incubation at 55 °C overnight, DNA was extracted using phenol:chloroform:isoamyl alcohol and precipitated with ethanol. Isolated DNA was dissolved in nuclease-free water and stored at −80 °C. The quality of the isolated DNA was detected using agarose gel electrophoresis.

### PCR amplification of the 16S rRNA gene

The primers used for amplification of the 16S rDNA V3 regions (230 bp) were as follows: V3F, 5′-ATTACCGCGGCTGCTGG-3′; and V3R: 5′-CGCCCGCCGCGCGCGGCGGGCGGGGCGGGGGCACGGGGGGCCTACGGGAGGCAGCAG-3′, which were synthesized by Shanghai Invitrogen Biotechnology Co., Ltd. The amplification was conducted in a 25 μL reaction containing 0.5 μL of template DNA (20 ng), 0.5 μL of Taq DNA polymerase (1.25 U), 2.5 μL of V3F and V3R primers (5 pmol), 2.5 μL of MgCl_2_ (50 mmol/L), 2.5 μL of Taq buffer (Mg^2+^free), 0.5 μL of dNTPs and 13.5 μL of ddH2O. The amplification conditions were as follows: 94 °C for 3 min; 30 cycles of 94 °C for 1 min, 65 °C for 1 min, and 72 °C for 1 min; and a final elongation step at 72 °C for 8 min. The amplification products were verified by 2% agarose gel electrophoresis and stored at −20 °C.

### Denaturing gradient gel electrophoresis (DGGE)

DGGE was carried out with the DCodeTM Universal Mutation Detection System (Bio-Rad, USA) according to the instructions of manufacturer. The conditions for separation were as follows: 40 μL of PCR products/lane, running at 150 V for 8 h in 1 × agarose gel electrophoresis buffer at 60 °C, and an 8% polyacrylamide gel with a denaturing gradient from 20 to 55%. After electrophoresis, the gel was stained with GelRed and then imaged with a grayscale scanner. Diversity analyses of the DGGE profiles were conducted using Quantity One (Bio-Rad, USA) and Biodap software. The diversity indices used included the Stripe number (S′), Shannon-Weaver Diversity Index (H′), Pielou Index (E) and Simpson index (D’) and the formulae for calculating them were as follows: H′ = −Σ(Ni/N) × ln(Ni/N), E = H′/lnS’, D’ = 1- Σ(Ni/N)^2^, where Ni was the grey value of single stripe, N was the grey value of all stripes, and S′ was the number of stripes.

### Establishment of an animal model of asthma

An animal model of asthma was established as previous described by Ikeda et al. [12] with slight modifications. At 36 days after birth, mice (*n* = 5) were intraperitoneally injected with 100 μL of OVA solution containing10 μg of OVA (Sigma, St. Louis, MO, USA) adsorbed to 4 mg of Al(OH)_3_ on days 0, 7 and 14 of the experiment. From day 19 to day 23, mice were sensitized with an aerosol of 10 Ml of 1% OVA for 30 min using an ultrasonic nebulizer every day. Using similar equipment and schedules, mice in the control group (*n* = 5) were injected intraperitoneally with a PBS-Al(OH)_3_ emulsion and then exposed to an aerosol of PBS without OVA.

On day 24, mice were killed by cervical dislocation within 24 h after the last OVA or PBS inhalation, and blood samples were collected by exsanguination following eyeball removal. After centrifugation at 4 °C, the serum was obtained and stored at −80 °C until analysis.

### Enzyme-linked immunosorbent assay (ELISA) for measurement of cytokines

Interleukin (IL)-4 and interferon (IFN)-γ in the serum were measured using commercially available ELISA kits (BD Biosciences, San Jose, CA, USA) following the recommend protocols of manufacturer.

### Histological analysis

Lungs were collected from the mice and then infused with 4% paraformaldehyde. Sections (5 μm thick) were embedded in paraffin and then stained with hematoxylin and eosin (H&E) to evaluate inflammatory changes. Pathological changes were observed by light microscopy at 200× magnification. Pathology was scored in a blinded fashion by three different individuals.

### Statistical analysis

Experimental data were expressed with mean ± SD. Differences among groups were analyzed by one-way ANOVA and further comparisons between two groups were evaluated by the post-hoc LSD test. All statistical analyses were performed using SPSS 13.0 (SPSS, Inc., Chicago, IL, USA). Differences were considered statistically significant at *P* values less than 0.05.

## Results

### Comparison of the intestinal flora of BALB/c mice in each group at different time period

**Fig. 1:**
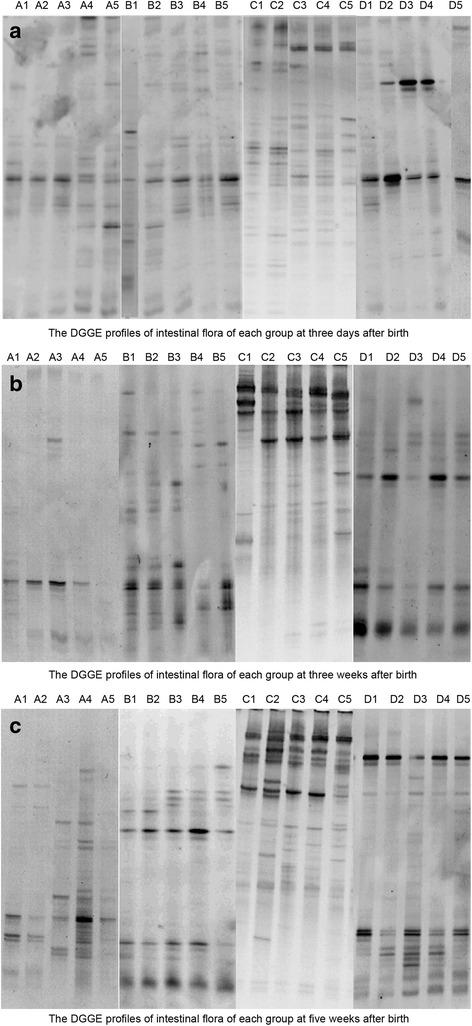
DGGE profile analysis. **a**: DGGE profiles showing the diversity of the intestinal flora of mice in the four groups at three days after birth. **b**: DGGE profiles showing the diversity of the intestinal flora of mice in the four groups at three weeks after birth. **c**: DGGE profiles showing the diversity of the intestinal flora of mice in the four groups at five weeks after birth. The greater the number of stripes, the greater the abundance of the intestinal flora. The lighter the stripes, the greater the number of species, which often represented dominant bacteria

As shown in Fig. 1, the number of different stripes in the DGGE profiles represented the diversity of the intestinal flora of mice in each group at different time period. The greater the number of stripes, the more abundant was the intestinal flora. The lighter the stripes were, the greater was the number of species, which often represented the dominant bacteria. As shown in Table 1, the S′, H′ and D’ of the intestinal microflora of BALB/c mice in group D were significantly higher than those of mice in groups A, B and C at three days and three weeks after birth (*P* < 0.05). However, there was no significant difference among groups A, B and C at three days after birth. In addition, the S′, H′ and D’ of intestinal microflora in group C were significantly higher than those of groups A and B at three weeks after birth (*P* < 0.05). At five weeks after birth, S′, H′ and D’ of intestinal microflora in groups C and D were significantly higher than those of groups A and B (*P* < 0.05), and there was no significant difference between groups C and D.

**Table 1:** The diversity analysis for DGGE profiles of different groups at different periods

Groups	S′	H′	E	D′
At three days after birth				
A (*n* = 5)	8.80 ± 1.643	2.013 ± 0.194	0.948 ± 0.193	0.849 ± 0.034
B (*n* = 5)	8.80 ± 1.924	2.126 ± 0.258	0.954 ± 0.0213	0.898 ± 0.047
C (*n* = 5)	9.60 ± 2.074	2.231 ± 0.224	0.958 ± 0.015	0.913 ± 0.049
D (*n* = 5)	30 ± 3.536^a,b,c^	3.396 ± 0.112^a^,^b,c^	0.997 ± 0.001^a^	0.968 ± 0.005^a^,^b,c^
At three weeks after birth				
A (*n* = 5)	10.00 ± 1.581	2.424 ± 0.217	0.998 ± 0.008	0.897 ± 0.017
B (*n* = 5)	12.20 ± 3.114	2.606 ± 0.246	0.993 ± 0.009	0.910 ± 0.025
C (*n* = 5)	40.80 ± 3.271^a^,^b^	3.690 ± .083^a^,^b^	0.996 ± 0.002	0.974 ± 0.002^a^,^b^
D (*n* = 5)	52.00 ± 4.000^a^.^b,c^	3.933 ± 0.078^a^,^b,c^	0.996 ± 0.004	0.980 ± 0.002^a^,^b^
At five weeks after birth				
A (*n* = 5)	22.60 ± 4.278	2.9376 ± 0.211	0.984 ± 0.002	0.946 ± 0.010
B (*n* = 5)	27.00 ± 8.367	3.2188 ± 0.344	0.989 ± 0.006	0.956 ± 0.016
C (*n* = 5)	55.60 ± 5.320^a^,^b^	4.00 ± 0.0732^a^,^b^	0.995 ± 0.004	0.982 ± 0.003^a^,^b^
D (*n* = 5)	59.80 ± 3.962^a^,^b^	4.074 ± 0.081^a^,^b^	0.996 ± 0.003^a^,^b^	0.982 ± 0.001^a^,^b^

*S′* the Stripe number, *H′* Shannon-Weaver Diversity Index, *E* Pielou Index, *D*′ Simpson index

^a^indicated significant difference compared with group A

^b^indicated significant difference compared with group B

^c^indicated significant difference compared with group C, *P* < 0.05

### The clinical manifestations of the OVA-induced asthma model

After OVA inhalation, the mice in all four groups showed symptoms of acute asthma attack, such as restlessness, wheezing, abdominal cramps, nodding breathing and shortness of breath. The mice in the control group did not show the afore-mentioned symptoms.

### The pathological changes in the OVA-induced asthma model

**Fig. 2:**
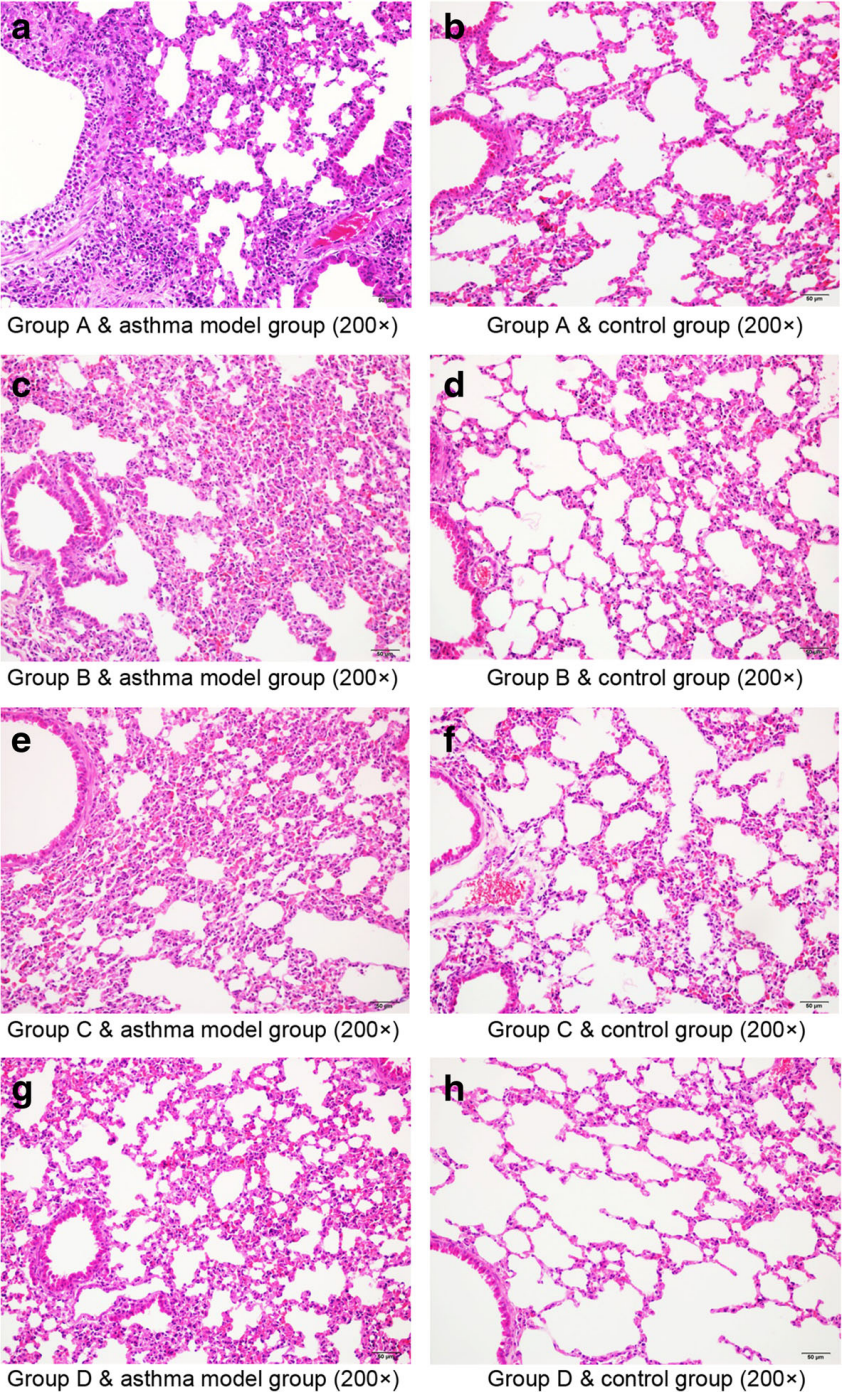
The pathological changes in the lungs of mice in different groups after OVA inhalation. **a**: Group A & asthma model group, **b**: Group A & control group, **c**: Group B & asthma model group, **d**: Group B & control group, **e**: Group C & asthma model group, **f**: Group C & control group, **g**: Group D & asthma model group and **h**: Group D & control group. The mice in the control group showed clear structures in the bronchus and alveoli with arranged neatly airway epithelial cells, intact lumen, no infiltration of inflammatory cells (magnification: × 200). The mice in the OVA-induced asthmatic model group showed disordered structures in the lung tissue, with widened alveolar septum, capillary dilatation, congestion, and a large number of inflammatory cell and neutrophil infiltrations (magnification: × 200)

As shown in Fig. 2, the mice in the control group showed clear structures in the bronchus and alveoli with arranged neatly airway epithelial cells, intact lumen, no infiltration of inflammatory cells. Compared with the control group, mice in the OVA-induced asthmatic model group showed disordered structures in the lung tissue, with widened alveolar septum, capillary dilatation, congestion, and a large number of inflammatory cell and neutrophil infiltrations. In addition, there were significant differences in the pathologic scores between the asthma model group and the control group (*P* < 0.05, Table 2). Moreover, the pathologic scores of group D were significantly lower than those of group A (*P* < 0.05, Table 2).

**Table 2:** Pathologic scores of lung samples in different groups

Groups	Pathological scores	*P* value
Group A & asthma model group (*n* = 5)	6.25 ± 2.78	0.00^a^
Group A & control group (*n* = 5)	0.75 ± 0.96	
Group B & asthma model group (*n* = 5)	5.00 ± 0.41	0.00^a^
Group B & control group (*n* = 4)	0.40 ± 0.55	
Group C & asthma model group (*n* = 4)	4.33 ± 0.58	0.00^a^
Group C & control group (*n* = 5)	0.88 ± 1.03	
Group D & asthma model group (*n* = 5)	3.50 ± 0.41^b,c^	0.00^a^
Group D & control group (*n* = 5)	0.92 ± 0.83	

^a^indicated significant difference compared with control group

^b^indicated significant difference compared with group A

^c^indicated significant difference compared with group B, *P* < 0.05

### Influence of the different environment on airway inflammation in the OVA-induced asthma model

We further investigated the influence of different environments (SPF or general) on airway inflammation in the OVA-induced asthma model (Table 3). In comparison with the control group, serum levels of IL-4 were significantly higher in OVA-induced asthmatic mice, whereas serum IFN-γ levels and the IFN-γ/IL-4 ratio were markedly lower (*P* < 0.05). However, no statistically significant differences were observed in the serum IL-4 levels in OVA-induced asthmatic mice between any two groups. Besides, serum IFN-γ levels and the IFN-γ/IL-4 ratio in the OVA-induced asthmatic mice of group D were significantly higher than those in group A (*P* < 0.05) and no significant difference was observed between other groups.

**Table 3:** The levels of IFN-γ and IL-4 in different groups

Groups	IFN-γ levels (pg/ml)	IL-4 levels (pg/ml)	IFN-γ/IL-4 (pg/ml)
Group A & asthma model group (*n* = 5)	57.31 ± 11.43^a^	8.24 ± 2.88^a^	7.80 ± 3.35^a^
Group A & control group (*n* = 5)	139.09 ± 22.66	4.47 ± 1.38	32.57 ± 7.08
Group B & asthma model group (*n* = 5)	62.30 ± 4.22^a^	6.62 ± 2.08^a^	10.09 ± 3.02^a^
Group B & control group (*n* = 4)	197.03 ± 91.52	3.88 ± 1.06	61.48 ± 55.76
Group C & asthma model group (*n* = 4)	75.36 ± 4.60^a^	6.63 ± 1.14^a^	11.60 ± 1.97^a^
Group C & control group (*n* = 5)	204.13 ± 68.77	3.66 ± 1.29	62.25 ± 36.45
Group D & asthma model group (*n* = 5)	96.38 ± 23.47^a,b^	7.74 ± 1.02^a^	12.69 ± 3.95^a,b^
Group D & control group (*n* = 5)	273.96 ± 50.82	4.01 ± 1.93	90.98 ± 66.95

^a^indicated significant difference compared with control group

^b^indicated significant difference compared with group A, *P* < 0.05

## Discussion

In the current study, we investigated the effect of early-life exposure to different microbial environments on gut microbial colonization in BALB/c mice. The results showed that the diversity of intestinal microflora in group D, which were reared in a general environment from the fetal period through childhood, was significantly higher than groups A, B and C, which spent some part of their early life in a SPF environment, at three days and three weeks after birth. The diversity of intestinal microflora in groups C and D was significantly higher than groups A and B at five weeks after birth. In addition, the pathologic scores of OVA-induced asthmatic mice in group D were significantly lower than those in group A, and the levels of serum IFN-γ and the IFN-γ/IL-4 ratio in OVA-induced asthmatic mice of group D were significantly higher than those in group A, implying that early-life exposure to a diverse microbial environment may prevent asthma development via regulating IFN-γ levels or the IFN-γ/IL-4 ratio. These findings suggest a key role for early-life exposure to diverse microbial environment in preventing asthma development and merit further consideration.

During early-life, the diversity and composition of the microbiota are influenced by a variety of environmental conditions, such as the rearing environment, exposure to stress and antibiotic use [13–16]. A previous study has showed that early-life airway bacterial colonization determines whether early-life exposure to diverse microbiota influences the risk of asthma development [17]. Moreover, alteration in early infant gut microbiota have been shown to have an important impact on the attenuation of allergic diseases [18]. A birth cohort study has confirmed the key roles of the diversity of environmental microbial exposure in the development of asthma [19]. In addition, early-life environmental variation is critical for gut microbial colonization in new-born piglets [20]. In our study, the S′, H′ and D’ of the intestinal microflora in group D were significantly higher than those in groups A, B and C at three days or three weeks after birth. At five weeks after birth, S′, H′ and D’ of intestinal microflora in groups C and D were significantly higher than those in groups A and B. It can therefore be speculated that exposure to different living environments in early life has a clear impact on gut microbial colonization.

Th1/Th2 imbalance has been shown to be a key contributor to asthma development [21]. It is reported that Astragalus membranaceus, a traditional Chinese herb, plays an inhibitory role in airway inflammation in a murine model of asthma via modulating Th1/Th2 immune balance [22]. Paeoniflorin can exert immunoregulatory effects to ameliorate asthma progression via modulation of the Th1/Th2 equilibrium [23]. Overproduction of Th2 cytokines, such as IL-4 and IL-5 which are released by Th2 cells, is shown to contribute to the pathophysiology of asthma [24]. IL-4 is also perceived as a marker of severe asthma [25]. Additionally, IFN-γ released by Th1 cells can also decrease Th2 immune responses in the airways, thus, it functions as a key regulator of allergic inflammation to prevent the symptoms of allergic asthma [26]. It has also been reported that balanced composition of the gut microbiome plays a crucial role in shifting the Th2 immune response towards Th1 to prevent asthma development [27, 28]. In our study, in comparison with control group, serum IL-4 levels were significantly higher in OVA-induced asthmatic mice, whereas serum IFN-γ levels and the IFN-γ/IL-4 ratio were obviously decreased, indicating that Th1/Th2 balance was disturbed in OVA-induced asthmatic mice. In addition, blocking of Tim-3 may be of therapeutic benefit for patients with allergic asthma by enhancing the Th1 cytokines response and down-regulating the Th2 cytokines response [29]. Our results showed that serum IFN-γ levels and the IFN-γ/IL-4 ratio in the OVA-induced asthmatic mice of group D were significantly higher than those in group A, suggesting that exposure to diverse microbial environments in early life induced a TH1 response. This also means that cohorts in group D have less airway inflammation from OVA exposure as their microbiome may shape the T-cell response towards a TH1 bias. Besides, the pathologic scores of mice in group D were significantly lower than those of mice in group A. Serum IFN-γ levels and the IFN-γ/IL-4 ratio of OVA-induced asthmatic mice in group D were significantly higher than those of mice in group A. Therefore, our results are consistent with previous findings and indicate that early-life exposure to diverse microbial environments may improve the Th1/Th2 balance, and thus reduce airway inflammation in asthma.

## Conclusions

In sum, our findings indicate that exposure to diverse microbial environments in early life produces a clear impact on gut microbial colonization in BALB/c mice. Additionally, the diversity of the intestinal flora in early life may prevent airway inflammation in asthma via regulating the Th1/Th2 balance. However, BALB/c mice produced by different providers may respond differently, which may influence the creditability of our results. Further experiments using more mice strains are still required to validate our findings.

## Abbreviations

DGGE: Denaturing gradient gel electrophoresis
H&E: Hematoxylin and eosin
IFN: Interferon
IL: Interleukin
SPF: Specific pathogen-free

## References

[1] Ahmad A, Shameem M, Husain Q (2012). Relation of oxidant-antioxidant imbalance with disease progression in patients with asthma. Ann Thorac Med.

[2] Lambrecht BN, Hammad H (2014). The immunology of asthma. Nat Immunol.

[3] Rath S, Alam S (2014). Psychology of Asthma.

[4] Martinez F (2007). Genes, environments, development and asthma: a reappraisal. Eur Respir J.

[5] Okada H, Kuhn C, Feillet H, Bach JF (2010). The ‘hygiene hypothesis’ for autoimmune and allergic diseases: an update. Clin Exp Immunol.

[6] Bisgaard H, Hermansen MN, Buchvald F, Loland L, Halkjaer LB, Bønnelykke K, Brasholt M, Heltberg A, Vissing NH, Thorsen SV (2007). Childhood asthma after bacterial colonization of the airway in neonates. N Engl J Med.

[7] Ege MJ, Mayer M, Normand AC, Genuneit J, Cookson WO, Braun-Fahrländer C, Heederik D, Piarroux R, von Mutius E; GABRIELA Transregio 22 Study Group.: Exposure to environmental microorganisms and childhood asthma. N Engl J Med 2011; 364:701-9. 10.1056/NEJMoa1007302 21345099

[8] Abrahamsson TR, Jakobsson HE, Andersson AF, Björkstén B, Engstrand L, Jenmalm MC (2012). Low diversity of the gut microbiota in infants with atopic eczema. J Allergy Clin Immunol.

[9] Bisgaard H, Li N, Bonnelykke K, Chawes BL, Skov T, Paludanmüller G, Stokholm J, Smith B, Krogfelt KA (2011). Reduced diversity of the intestinal microbiota during infancy is associated with increased risk of allergic disease at school age. J Allergy Clin Immunol.

[10] Thavagnanam S, Fleming J, Bromley A, Shields MD, Cardwell CR (2008). A meta-analysis of the association between caesarean section and childhood asthma. Clin Exp Allergy.

[11] Pospiech A, Neumann B (1995). A versatile quick-prep of genomic DNA from gram-positive bacteria. Trends Genet.

[12] Ikeda Y, Kaneko A, Yamamoto M, Ishige A, Sasaki H (2002). Possible involvement of suppression of Th2 differentiation in the anti-allergic effect of Sho-seiryu-to in mice. Jpn J Pharmacol.

[13] Inman CF, Haverson K, Konstantinov SR, Jones PH, Harris C, Smidt H, Miller B, Bailey M, Stokes C (2010). Rearing environment affects development of the immune system in neonates. Clin Exp Immunol.

[14] Mulder IE, Schmidt B, Lewis M, Delday M, Stokes CR, Bailey M, Aminov RI, Gill BP, Pluske JR, Mayer CD (2011). Restricting microbial exposure in early life negates the immune benefits associated with gut colonization in environments of high microbial diversity. PLoS One.

[15] Schmidt B, Mulder IE, Musk CC, Aminov RI, Lewis M, Stokes CR, Bailey M, Prosser JI, Gill BP, Pluske JR (2011). Establishment of normal gut microbiota is compromised under excessive hygiene conditions. PLoS One.

[16] Cho I, Yamanishi S, Cox L, Methé BA, Zavadil J, Li K, Gao Z, Mahana D, Raju K, Teitler I (2012). Antibiotics in early life alter the murine colonic microbiome and adiposity. Nature.

[17] Beigelman A, Weinstock GM, Bacharier LB (2014). The relationships between environmental bacterial exposure, airway bacterial colonization, and asthma. Curr Opin Allergy Clin Immunol.

[18] Fooladi AA, Khani S, Hosseini HM, Mousavi SF, Aghdam EM, Nourani MR (2013). Impact of altered early infant gut microbiota following breastfeeding and delivery mode on allergic diseases. Inflamm Allergy Drug Targets.

[19] Karvonen AM, Hyvärinen A, Rintala H, Korppi M, Täubel M, Doekes G, Gehring U, Renz H, Pfefferle PI, Genuneit J (2014). Quantity and diversity of environmental microbial exposure and development of asthma: a birth cohort study. Allergy.

[20] Schokker D, Zhang J, Zhang LL, Vastenhouw SA, Heilig HG, Smidt H, Rebel JM, Smits MA (2014). Early-life environmental variation affects intestinal microbiota and immune development in new-born piglets. PLoS One.

[21] Kuo ML, Huang JL, Yeh KW, Li PS, Hsieh KH (2001). Evaluation of Th1/Th2 ratio and cytokine production profile during acute exacerbation and convalescence in asthmatic children. Ann Allergy Asthma Immunol.

[22] Chen SM, Tsai YS, Lee SW, Liu YH, Liao SK, Chang WW, Tsai PJ (2014). Astragalus membranaceus modulates Th1/2 immune balance and activates PPARγ in a murine asthma model. Biochem Cell Biol.

[23] Zhang T, Yang Z, Yang S, Du J, Wang S (2015). Immunoregulatory effects of paeoniflorin exerts anti-asthmatic effects via modulation of the Th1/Th2 equilibrium. Inflammation.

[24] Busse WW, Lemanske RF (2001). Asthma. N Engl J Med.

[25] Rogala B, Bozek A, Gluck J, Jarzab J (2015). Prevalence of IgE-mediated allergy and evaluation of Th1/Th2 cytokine profiles in patients with severe bronchial asthma. Adv Dermatol Allergol.

[26] Lack G, Bradley KL, Hamelmann E, Renz H, Loader J, Leung DY, Larsen G, Gelfand EW (1996). Nebulized IFN-gamma inhibits the development of secondary allergic responses in mice. J Immunol.

[27] Heederik D, Mutius EV (1996). Does diversity of environmental microbial exposure matter for the occurrence of allergy and asthma?. Natl Tax J.

[28] Hörmannsperger G, Clavel T, Haller D (2012). Gut matters: microbe-host interactions in allergic diseases. J Allergy Clin Immunol.

[29] Tang F, Wang F, An L, Wang X (2015). Upregulation of Tim-3 on CD4(+) T cells is associated with Th1/Th2 imbalance in patients with allergic asthma. Int J Clin Exp.

